# Case Report: Successful Long-Term Management of a Low-Birth Weight Preterm Infant With Compound Heterozygous Protein C Deficiency With Subcutaneous Protein C Concentrate Up to Adolescence

**DOI:** 10.3389/fped.2021.591052

**Published:** 2021-09-28

**Authors:** Johannes Pöschl, Wolfgang Behnisch, Bernd Beedgen, Navina Kuss

**Affiliations:** ^1^Department of Neonatology, Heidelberg University Children's Hospital, Heidelberg, Germany; ^2^Department of Pediatric Oncology, Hematology and Immunology, Heidelberg University Children's Hospital, Heidelberg, Germany

**Keywords:** congenital protein C deficiency, protein C concentrate, preterm infant, subcutaneous infusion, replacement therapy

## Abstract

Homozygous/compound heterozygous forms of congenital protein C deficiency are often associated with severe antenatal and postnatal thrombotic or hemorrhagic complications. Protein C deficiency frequently leads to severe adverse outcomes like blindness and neurodevelopmental delay in children and may even lead to death. The most widely used long-term postnatal treatment consists of oral anticoagulation with vitamin K antagonists (e.g., warfarin), which is supplemented with protein C concentrate in acute phases. Subcutaneous infusions have been described in infants mostly from 2 months of age after severe postnatal thrombosis, but not in newborns or premature infants without thromboembolism. We report the first case of a compound heterozygous protein C-deficient preterm infant, born at 31+5 weeks of gestation to parents with heterozygous protein C deficiency (protein C activity 0.9% at birth). We focus on both prenatal and perinatal management including antithrombotic treatment during pregnancy, the cesarean section, and continuous postnatal intravenous and consecutive subcutaneous therapy with protein C concentrate followed by a change of therapy to direct oral anticoagulants (DOACs) (apixaban). We report successful home treatment with subcutaneous protein C concentrate substitution overnight (target protein C activity >25%) without complication up to 12.5 years of age. We propose that early planned cesarean section at 32 or preferably 34 weeks of gestation limits potential maternal side effects of anticoagulation with vitamin K antagonists and reduces fetal thromboembolic complications during late pregnancy. Intravenously administered protein C and early switch to subcutaneous infusions (reaching about 3 kg body weight) resulted in sufficient protein C activity and has guaranteed an excellent quality of life without any history of thrombosis for 13 years now. In older children with protein C deficiency, as in our case, DOACs could be a new therapeutic option.

## Introduction

In neonates with severe congenital protein C deficiency, the homozygous/compound heterozygous form is often associated with retinal vessel thrombosis that results in vitreous hemorrhage and retinal detachment, leading to partial or complete blindness (1, 2). Additional severe complications include antenatal cerebral vein thrombosis during late pregnancy (hypothetically during the last 6 weeks of gestation) or after birth with severe purpura fulminans (1–5). While the incidence of heterozygous congenital protein C deficiency is 1 in 200–500 births (5), the homozygous state is extremely rare between 1 in 250,000 (6) and up to 1 in 4 million (5, 7, 8). To date, there have been more than 160 protein C mutations described (9). We report the case of a compound heterozygous preterm infant with protein C deficiency, born in 2006 at 31+5 weeks of gestation to parents with heterozygous protein C deficiency and clinical follow-up until 2019. With two heterozygous parents, the child has a 25% risk of being compound heterozygous for protein C deficiency that equals functional homozygosity if each single PROC gene mutation causes thrombosis (8).

The most widely used long-term treatment is using vitamin K antagonists (warfarin) and protein C concentrate in the acute phase of complications (2, 10). Subcutaneous infusions in children are described mostly from the age of 2 months after severe postnatal thrombosis but not in newborns or premature infants without thromboembolism (11–15). We focus on perinatal management including antithrombotic treatment during pregnancy, the cesarean section, and the daily postnatal intravenous followed by subcutaneous therapy with protein C concentrate.

## Case Report

A 24-year-old woman (gravida II, para 0) was referred at 21 weeks of gestation for genetic counseling including amniocentesis, as both unrelated parents suffered from heterozygous protein C deficiency. The woman had a history of thrombosis and lung embolism 4 years previously that had resulted in therapy with a vitamin K antagonist (phenprocoumon). During early pregnancy, the mother was treated with low-molecular weight heparin (5,000 IU dalteparin q24h). Genetic testing was initiated as part of the pregnancy counseling of the parents and revealed paternal heterozygous protein C mutation as well as a heterozygous factor V Leiden mutation despite no history of thrombosis up to this time (Table 1). Thrombosis or mutations in the PROC gene in other first-degree relatives of the parents were also not known. Amniocentesis was preceded by detailed counseling regarding the risks of pregnancy, the probability of disease, and the risk of mortality and morbidity of the child.

**Table 1:** Disease-related family history and selected laboratory results.

**Family member**	**Protein C activity (%)**	**Genetic mutation PROC gene (2q13–q14)**	**History of thrombosis**	**Anticoagulatory therapy**
Patient	0.9^*^	Exon 9 Val325Met-mutation	No	Protein C concentrate from birth to 12.5 years of age; since then Apixapan
		Exon 9 Met343Ile-mutation		
Mother	43.0	Exon 9 Val325Met-mutation	Yes^°^	Early pregnancy low molecular heparin until 23+6 weeks of gestation; before and after phenprocoumon
Father	52.0	Exon 9 Met343Ile-mutation^$^	No	No

* *Protein C activity at birth before treatment with protein C concentrate*.

$ *Additional heterozygous factor V Leiden mutation*.

° *Deep vein thrombosis and lung embolism before pregnancy*.

The results of the amniocentesis showed a heterozygous mutation in codon 325 (GTG□ATG) in exon 9 of the PROC gene with exchange of the amino acid valine against methionine (val325met-mutation) (maternal mutation) and heterozygous mutation in codon 343 (ATG□ATA) in exon 9 of the PROC gene with exchange of the amino acid methionine against isoleucine (met343ile-mutation) (paternal mutation). The fetus had inherited both mutations of his parents resulting in compound heterozygous protein C deficiency (Table 1).

The val325met-mutation has been described in patients with clinically apparent protein C deficiency (9). In the absence of expression studies, there is only indirect evidence for a resulting protein C deficiency. The met343ile-mutation however is generally known to lead to protein C deficiency (16).

After receiving the results of the amniocentesis, we performed weekly ultrasound examinations of the fetus as well as a fetal magnetic resonance imaging (MRI) at 28 weeks of gestation, which did not indicate any abnormalities.

As opposed to low-molecular weight heparin, which does not cross the placental barrier in significant amounts, treatment with vitamin K antagonists generates sufficient thrombosis prophylaxis for both mother and fetus (17, 18). After informed consent, we started oral anticoagulation with phenprocoumon the first days in overlap with heparin after 23+6 weeks of gestation to prevent intrauterine retinal detachment and fibrosis of the retinal membrane and cerebral thrombosis of the fetus. For the remaining pregnancy, the mother and the fetus were closely monitored as inpatients.

To achieve maximum safety for both mother and fetus, primary cesarean section was scheduled for 32 weeks of gestation but performed at 31+5 weeks of gestation due to premature rupture of membranes. The effects of phenprocoumon were antagonized several hours before surgery by substitution of 3,500 IU of PPSB (prothrombin complex, containing the factors II, VII, IX, and X) with 500 IU antithrombin and one platelet concentrate.

A male preterm infant was delivered (1,680 g, APGAR 7/7/8) and admitted to the neonatal intensive care unit (NICU) at Heidelberg University (level III perinatal center).

Postnatally, the infant displayed a normal ophthalmic examination, normal ultrasound of the brain, and no signs of purpura. The protein C activity was 0.9% (12–44%); D-dimers, 0.27 mg/L; elevated international normalized ratio (INR), 2.02; partial thromboplastin time, 55.7 s (27–75 s); antithrombin activity, 31% (14–62%); hemoglobin level, 14.6 g dl^−1^; white blood cell count, 5,900 μl^−1^; platelet count, 237,000 μl^−1^ (19).

The newborn presented with respiratory distress syndrome. Initially, respiratory support by continuous positive airway pressure (CPAP) was delivered, at the age of 12 h, secondary intubation and surfactant application were performed. On the fifth day, the boy could be extubated successfully under caffeine therapy. He received the recommended neonatal vitamin K therapy right after birth as well as on days 5 and 28 of life.

Immediately after birth, 120 IU/kg protein C concentrate (Ceprotin®, Baxter Co., Germany) was infused over 10 min, *via* a peripheral venous line, followed by 60 IU/kg every 6 h, aiming at >30% physiological protein C activity (5, 14, 15, 19, 20) (Table 2). Sufficient activity levels were reached during the first day of life, and protein C concentrate was daily infused with increasing intervals from 6 over 8 to 12 h until the infant's weight was 3 kg. At 38 corrected weeks (days 50–51), subcutaneous infusion was started and overlapped with intravenous infusion for 2 days. Subcutaneous infusion (1 ml/h, i.e., 100 IU/ml) was carried out using an infusion set (DISETRONIC tender, Burgdorf, Switzerland) with a PEGA® plus box infusion pump (PEGASUS, Kiel, Germany). The subcutaneous needle (25G, initial 9 mm, later on 17 mm s.c. MediPro) was changed once or twice a week. The puncture site on the abdomen was rotated clockwise.

**Table 2:** Course of treatment.

**Age**	**Interval of infusions (h)**	**Protein C concentrate (IU/kg/day)**	Protein C activity means (minimum–maximum) (%)^*^	**D-Dimers minimum–maximum (mg/L)**
At birth	None	None	0.9	0.27
1 day	6	180 i.v.	30.6	0.45
2–5 days	6	240 i.v.	32.5 (24.8–43.8)	0.21–0.33
6–7 days	8	240 i.v.	41.1 (37.5–49.9)	0.21–0.24
8–21 days	12	170–200 i.v.	31.0 (21.8–39.1)	0.21–0.26
22–45 days	12–13	210–240 i.v.	33.5 (16.8–56.8)	0.10–0.36
45–50 days	12	200 i.v.	33.4 (13.2–39.4)	0.05–0.25
50–57 days	24	154–160 s.c.^°^	26.8 (18.9–36.9)	0.07–0.33
58–59 days	40–42	300 s.c.^°^	26.1 (25.4–27.8)	0.06–0.07
60–73 days	24	125–148 s.c.^°^	25.2 (13.3–33.1)	0.08–0.12
10 months	24	100 s.c.^°^	42.0 (30.2–57.8)	<0.05^♢^
1.0–12.5 years	24	1,500 IU^∧^ independent of body weight	50.2 (27.0–94.6)^□^	0.19–0.50
12.5–13.5 years^$^	None	None	No mean value available (5.0–10.0)	<0.05^♢^

* *Functional protein C activity was measured with an automated chromogenic assay. Indicated are the trough levels*.

° *Infusion rate 1 ml/h [100 IU/ml]*.

∧ *Infusion rate 1.25 ml/h [100 IU/ml]*.

□ *Because the determination of protein C activity was performed in the context of outpatient controls, the time intervals between blood sampling and the end of the subcutaneous protein C infusion differ for technical reasons. Minimal trough level of protein C activity 27.0%*.

♢ *values below the measurable range*.

$ *Since the age of 12.5 years, the therapy was changed to apixaban 3 × 5 mg/day at a body weight of 50–59 kg. i.v., intravenous administration; s.c., subcutaneous administration*.

An attempt to prolong infusion intervals to every other day by increasing the amount of protein C concentrate from 154 to 300 IU/kg and the corresponding infusion time from 5 to 10 h (infusion rate of 1 ml/h) on the day of infusion (tested on days 58–59) did not result in sufficient basal protein C activity >30% after 42 h. Therefore, daily infusion was chosen for further treatment.

The infant was discharged on day 73 (corrected, 42 weeks; weight, 4.1 kg) with maintenance therapy of 1,000 IU/day protein C concentrate (243 IU/kg) administered by subcutaneous infusion pump for 10–12 h overnight (infusion rate 1 ml/h). Up to the date of discharge, the infant received a total of 34,560 IU of protein C.

At the age of 1 year, we stopped protein C substitution under prophylaxis of low-molecular weight heparin therapy (anti Xa activity 0.4–0.8) to test for baseline activity and half-life due to reports on maturation of protein C function over time (21). After infusion of 1,000 IU protein C concentrate, protein C activity decreased from 75.4 to 38.7% after 16 h and to 24.8% after 38 h. After that, subcutaneous protein C substitution was reinitiated. The subcutaneous application half-life was 17 h on this day.

During the following years, no further baseline or half-life tests were performed. Treatment was monitored by regular measurements of protein C activity under substitution and the absence of thromboembolic events. D-dimers and fibrinogen, which are often used for monitoring, were measured regularly (5). Except for occasional minor reddening of the skin at the injection site, which disappeared quickly after the needle was changed, there were no side effects associated with subcutaneous protein C substitution. Investigations to detect the occurrence of protein C inhibitory alloantibodies were performed at 1 year of age. After that, there was no need for regular testing. The protein C activities were always within the target range under constant dosage. The child received the first regular vaccination during the initial hospitalization and received all other vaccinations according to the recommendations of the Standing Committee on Vaccination (STIKO Germany). Because infections are known to be a trigger for thromboembolic events, protein C activity (trough and peak activity levels) were immediately monitored at the onset of fever or other signs of infection until recovery. A prophylactic increase of the daily protein C dose was not performed. In the context of a febrile obstructive bronchitis episode at the age of 10 months, the protein C activity measured at hospital admission was 38.4% (peak level), only slightly above the target range, so that the daily dose had to be increased from 100 to 150 IU/kg/day over 5 days. No bleeding or thrombotic events occurred during this episode; D-dimers, prothrombin, activated partial thromboplastin time (APTT), thrombin clotting time, and fibrinogen concentration were within physiological ranges at all times.

As of now, the child is 13 years old (weight, 59 kg) without any thrombotic events. Under subcutaneous substitution of 1,500 IU/day protein C (last 30 IE/kg) during a 10–12-h period overnight, baseline protein C activity of 25% could be reached. During the last months of subcutaneous therapy, pump alarms due to needle dislocation or technical problems increasingly disturbed the sleep of the young boy. The adolescent was increasingly dissatisfied with the current treatment regime, and compliance was likely to be compromised. The available treatment options, including continuation of the current therapy, the use of vitamin K antagonists, and the administration of direct oral anticoagulants (DOACs) as experimental individual therapy, were discussed with the parents and the adolescent, taking into account all risks, side effects, and benefits of the respective therapy. Together with the family, we reached informed consent to change therapy to DOAC (apixaban 3 × 5 mg/day). The switch in therapy was carried out under close monitoring (clinical and laboratory values) especially in trigger situations such as infections.

Up to the time of this report, ophthalmic examinations, 3D time-of-flight (TOF) MR angiography at 3.0 T of the circle of Willis, and MRIs of the brain were normal, and no episodes of purpura fulminans occurred. The boy has developed regularly and actively participates in sports.

## Discussion

To our knowledge, this is the first case of a prenatally diagnosed compound heterozygous fetus with protein C deficiency that was successfully treated prenatally and postnatally without development of any thrombotic complication until more than 13 years of life.

In the present case, early diagnosis in the fetus allowed adequate anticoagulant therapy of mother and child during pregnancy and immediate initiation of protein C substitution postnatally (5, 22). Severe thromboembolic events, often already observed intrauterine, could thus be avoided (1, 5). In the case of heterozygous protein C deficiency of the mother, genetic counseling of the parents and genetic screening of the father and of the fetus seem to be important components of an adequate therapy. This is particularly relevant if the parents decide to continue the pregnancy. Regular imaging studies of the fetus are necessary to detect thrombosis or vitreal bleeding and were performed weekly in the patient described here until delivery in the form of ultrasonography and a fetal MRI.

During pregnancy, the only treatment option generating sufficient maternal and fetal anticoagulation with an acceptable and well-defined risk profile is oral vitamin K antagonists (23, 24). Indeed, treatment with phenprocoumon resulted in significant elevation of the fetal INR as confirmed after delivery and can be viewed as effective in therapeutically inhibiting fetal coagulation. Maternal treatment with vitamin K antagonist was effectively antagonized for early planned cesarean section without influencing the fetus anticoagulant therapy at any point. Since early intrauterine thrombosis of the fetus may occur during the last 6–8 weeks of pregnancy, the planned delivery at 32 weeks of gestation was a compromise between risks of preterm birth and the increasing risks for intrauterine thrombosis with blindness and cerebral hemorrhage. As the limited available data suggest a low risk for intrauterine cerebral hemorrhage or placental abruption between 32 and 34 weeks of gestation under adequate maternal anticoagulant therapy, we propose a planned delivery at 34 weeks of gestation to minimize possible side effects for the late preterm.

In severe congenital protein C deficiency, the treatment options include fresh frozen plasma (FFP) and protein C concentrate in the acute phase (5, 10, 20, 22, 25, 26). The American College of Chest Physicians (ACCP) guidelines for antithrombotic therapy in symptomatic neonates and children recommend acute treatment with either 10–20 ml/kg FFP every 12 h or protein C concentrates at 20–60 IU/kg every 6 h until resolution of clinical lesions. Due to the low number of cases, there are no clear guidelines regarding dose and frequency of administration in neonates with compound heterozygous protein C deficiency. Li et al. (8) describe that almost 30 cases of congenital protein C deficiency in newborns have been reported globally. But some review articles and case reports support this approach in the last years (2, 5, 8, 22).

The most widely used long-term therapy consists of vitamin K antagonists for anticoagulation with inherent difficulties in maintaining sufficient INR levels in infants and the long-term effect on bone matrix protein in growing children (10). In the present case with a very low initial protein C activity of 0.9% (age-specific normal values 12–44%), sufficient protein C activities could be achieved by administration of 120 IU/kg protein C concentrate immediately after birth and continuous careful dose adjustment according to the obtained trough protein C activities (2, 19). Since long-term intravenous applications require peripheral and central lines with high risk for phlebitis, sepsis, and catheter thrombosis, the preterm infant was substituted with protein C for 15 min intravenously *via* peripheral line only until 3 kg of weight and corrected age of 38 weeks of gestation. Then, the route of administration was switched to subcutaneous infusion over 5 or 10 h resulting in nearly 40% reduction of needed protein C concentrate compared to short time intravenous infusion (Table 2). Other cases reported subcutaneous infusion in infants starting older than 1–9 months after postnatal severe thrombosis and after intravenous protein C substitution. All of them were blind or had severe visual impairment, except our patient (11–15). Our case indicates that switching intravenous to subcutaneous administration at term is effective when the infants have enough subcutaneous adipose tissue. Moreover, a 10-h infusion time was superior to 5 h to reach sufficient protein C activity levels.

For long-term therapy after discharge, we recommended a basal protein C activity of ~25%, which was reached independent of the weight gain with a subcutaneous infusion overnight with a daily dose of 500 IU protein C concentrate from 2 to 9 months, 1,000 IU protein C up to 16 months, and 1,500 IU protein C thereafter. Despite the significant body weight gain over 10 years, the protein C distribution volume and a stable protein C activity >25% were reached until adolescence. We based the target value of >25% on the standard values given in the literature for patients with mild protein C deficiency aged over 1 year up to adulthood (21).

At the age of 12.5 years, due to more frequent problems with the pump system and the risk of reduced patient compliance, we decided together with the family to start an off-label therapy with apixaban (DOAC) 3 × 5 mg/day. DOACs might control protein C deficiency in a more stable manner than vitamin K antagonists due to more constant drug levels and fewer side effects and because the former suppress the thrombotic tendency without reducing protein C and protein S production (27). However, little is known about the use of DOACs for pediatric patients and patients with protein C deficiency (27–29).

## Conclusion

Prenatal diagnosis of congenital compound heterozygous protein C deficiency can be safely managed by maternal treatment with vitamin K antagonists during the last trimester. Early planned cesarean section at 32+0 or better at 34 weeks of gestation limits potential maternal side effects of vitamin K antagonists and avoids the increasing fetal risk to develop severe intrauterine thromboembolic complications during late pregnancy. After delivery, intravenously administered protein C concentrate and a switch to subcutaneous infusion after reaching 3 kg body weight resulted in sufficient protein C activity >25%. Subcutaneous infusions have enabled the family to perform home treatment for 12.5 years with excellent quality of life for the child without visual or neurological impairment and without any history of thrombosis. The switch to off-label adixapan therapy has so far been tolerated without side effects and thromboembolic complications. To assess the potential of DOACs to serve as a novel therapeutic option in older children with protein C deficiency, further studies are warranted.

## Data Availability Statement

The original contributions presented in the study are included in the article/supplementary material, further inquiries can be directed to the corresponding author/s.

## Ethics Statement

Ethical review and approval was not required for the study on human participants in accordance with the local legislation and institutional requirements. Written informed consent to participate in this study was provided by the participants' legal guardian/next of kin. Written informed consent was obtained from the minor(s)' legal guardian/next of kin for the publication of any potentially identifiable images or data included in this article.

## Author Contributions

JP analyzed and interpreted data, wrote the manuscript mainly, and managed and supervised the treatment of the patient. WB and BB were significantly involved in the treatment of the patient, managed the 10 years' follow-up, interpreted data, and revised the manuscript. NK acquired data, was significantly involved in the treatment, and wrote parts of the manuscript. All authors have met the Frontiers in Pediatrics authorship requirements, read and approved the final version to be published.

## Conflict of Interest

The authors declare that the research was conducted in the absence of any commercial or financial relationships that could be construed as a potential conflict of interest.

## Publisher's Note

All claims expressed in this article are solely those of the authors and do not necessarily represent those of their affiliated organizations, or those of the publisher, the editors and the reviewers. Any product that may be evaluated in this article, or claim that may be made by its manufacturer, is not guaranteed or endorsed by the publisher.
